# Cellular and Molecular Aspects of the β-*N*-Methylamino-l-alanine (BMAA) Mode of Action within the Neurodegenerative Pathway: Facts and Controversy

**DOI:** 10.3390/toxins10010006

**Published:** 2017-12-22

**Authors:** Nicolas Delcourt, Thomas Claudepierre, Thomas Maignien, Nathalie Arnich, César Mattei

**Affiliations:** 1 Toulouse NeuroImaging Centre (ToNIC), INSERM 1214, Poison Control Centre, Toulouse-Purpan University Hospital, 31059 Toulouse, France; nicolas.delcourt@inserm.fr; 2 UR AFPA—INRA USC 340, EA 3998, Équipe Qualité de l’Alimentation et Vieillissement (QUALIVIE), Université de Lorraine, 54500 Vandoeuvre-les-Nancy, France; thomas.claudepierre@univ-lorraine.fr; 3 ANSES—French Agency for Food, Environmental and Occupational Health & Safety, Direction de l’Evaluation des Risques, 14 Rue Pierre et Marie Curie, 94701 Maisons-Alfort, France; thomas.maignien@anses.fr (T.M.); nathalie.arnich@anses.fr (N.A.); 4 UMR CNRS 6214, INSERM U1083, Mitovasc Institute, Angers University, 49045 Angers, France

**Keywords:** BMAA, neuromelanin, glutamate receptor, excitotoxicity, neurodegenerative disorders, intracellular calcium

## Abstract

The implication of the cyanotoxin β-*N*-methylamino-l-alanine (BMAA) in long-lasting neurodegenerative disorders is still a matter of controversy. It has been alleged that chronic ingestion of BMAA through the food chain could be a causative agent of amyotrophic lateral sclerosis (ALS) and several related pathologies including Parkinson syndrome. Both in vitro and in vivo studies of the BMAA mode of action have focused on different molecular targets, demonstrating its toxicity to neuronal cells, especially motoneurons, and linking it to human neurodegenerative diseases. Historically, the hypothesis of BMAA-induced excitotoxicity following the stimulation of glutamate receptors has been established. However, in this paradigm, most studies have shown acute, rather than chronic effects of BMAA. More recently, the interaction of this toxin with neuromelanin, a pigment present in the nervous system, has opened a new research perspective. The issues raised by this toxin are related to its kinetics of action, and its possible incorporation into cellular proteins. It appears that BMAA neurotoxic activity involves different targets through several mechanisms known to favour the development of neurodegenerative processes.

## 1. Introduction

**Figure 1:**
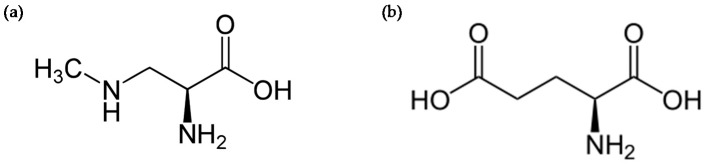
Chemical structure of β-*N*-methylamino-l-alanine (BMAA) (**a**), and glutamate (**b**). From Wikimedia commons.

β-*N*-methylamino-l-alanine (BMAA) is a non-canonical amino acid (Figure 1a), postulated to originate from various phytoplankton taxa including strains of freshwater cyanobacteria [1,2], marine diatoms [3,4] and dinoflagellates (*Gymnodinium catenatum*) [5]. The toxin community has dedicated considerable attention to BMAA because of its putative implication in slow-developing neurodegenerative diseases, notably the amyotrophic lateral sclerosis/Parkinson-dementia complex (ALS/PDC) [6]. However, the epidemiological link between human exposure to the toxin and the development of such pathologies has still to be made. Indeed, no other natural toxin has been shown to be the causative factor of a neurodegenerative disorder to date. The possible involvement of the consumption of shellfish contaminated by BMAA in the occurrence of neurodegenerative diseases may arise as one of the causative factors. Among the indigenous population of the island of Guam, suspected cases of ALS/PDC observed in the 1950s are claimed to have been caused by the accumulation of BMAA in the food chain from seeds and fruits of a cycad tree (*Cycas micronesica*) with cyanobacterial root symbionts [1]. In addition, the traditional consumption of bats feeding on cycad seeds by this indigenous population has been proposed as the main vector of exposure [7,8]. The causal relationship between BMAA and ALS has been claimed for a long time, with the cohort of Guam, but more recent studies have pointed to BMAA in seafood (e.g., bivalves and crustacean) as a factor in the chronic development of several neurodegenerative pathologies [9], with further confirmations of the BMAA contamination of the implicated shellfish by recent and robust analytical methods [10,11,12]. BMAA was analyzed in post-mortem brain samples from American patients who had been diagnosed with neurodegenerative illnesses, namely Alzheimer’s disease (AD), ALS, Huntington’s disease, versus neurological disease-free controls [13]. Although the estimation of BMAA amounts is a matter of controversy, especially because of the technique used to monitor its brain level, a large body of evidence is in the direction of the presence of bound BMAA (or one of its isomers 2,4-diamino butyric acid, DAB; β-amino-*N*-methyl-alanine, BAMA and *N*-(2-aminoethyl)glycine, AEG) in ALS and AD brains. However, a recent study reports the absence of BMAA in brains of 20 patients who died of AD [14]. This hypothesis must, therefore, be relativized at least for AD. BMAA was detected in the cerebrospinal fluid (CSF) collected ante-mortem from a patient with ALS, which suggests the ability of the toxin to cross the blood-brain barrier (BBB) [15]. As the frequent and regular consumption of contaminated shellfish may be a common risk factor for sporadic ALS, it makes the toxin BMAA one of the possible etiological agents of the disease. We review the mode of action of BMAA at the cellular and molecular levels in the central nervous system (CNS), in parallel with its putative involvement in long-lasting neurodegenerative disorders. The putative incorporation of the toxin in intracellular proteins is considered in different hypotheses presented. These hypotheses are not mutually exclusive.

## 2. BMAA-Induced Excitotoxicity through Glutamate Receptors

**Figure 2:**
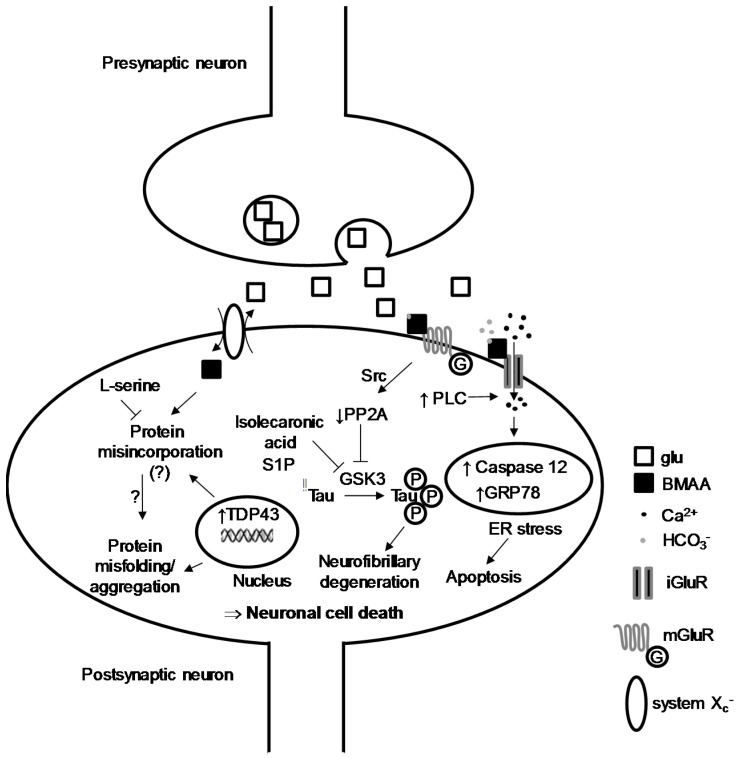
Molecular aspects of BMAA-induced neurodegeneration mechanisms. At a glutamatergic synapse, BMAA toxin binds to ionotropic (iGluR) and metabotropic (mGluR) receptors. Their activation leads to a significant increase in intracellular Ca^2+^, directly via iGluR and indirectly via mGluR (PLC signaling). This Ca^2+^_i_ increase promotes ER stress and cell apoptosis. In parallel, inhibition of PP2A induces hyperphosphorylation of Tau protein, which produces tangle degeneration. Finally, the X_c_^−^ system is a cystine/glu cotransport that is highjacked by BMAA to penetrate the postsynaptic neuron. Once in the cytoplasm, the toxin is likely to insert into the neosynthesized cellular proteins and to cause the aggregation of misfolded proteins that leads to neuronal death. GRP78: 78 kDa glucose-regulated protein; GSK-3: glycogen synthase kinase-3; iGluR: ionotropic glu receptors; mGluR: metabotropic glu receptors; PLC: phospholipase C; PP2A: protein phosphatase 2A; S1P: sphingosine-1-phosphate; Tau: Tubulin-associated unit; Src: proto-oncogene tyrosine-protein kinase; TDP-43: TAR DNA-binding protein 43.

One of the first and ongoing hypotheses related to the development of ALS is an excessive release of the neurotransmitter glutamate (glu, Figure 1b) within the central glutamatergic networks [16]. This over-stimulation of postsynaptic neurons typically leads to neuronal death, a phenomena termed excitotoxicity. The cascade of cellular and molecular events leading to ALS is, however, not fully characterized, but the excitotoxicity induced by glu seems to be at the core of cell dysfunctions leading to neurodegeneration. The main assumptions about the mode of action of BMAA have emerged from in vitro toxicological studies carried out on different cellular models, notably rodent, leech and human cell lines. Its ability to induce excitotoxicity is the mechanism most often proposed [17]. BMAA was first tested at relatively high concentrations (1 mM to 3 mM) on primary cultures of cortical mouse neurons [18], where it induces neurotoxic and neuroexciting effects if physiological concentrations (10 mM) of HCO_3_^−^ are added to the extracellular medium. HCO_3_^−^ is believed to interact with the toxin to provide a carboxylic group necessary for the interaction with glu receptors [18]. This dependence on HCO_3_^−^ has been found in other in vitro studies, including non-neuronal cells such as glial cells, but again at high concentrations of toxin, 0.5 mM to 3 mM [17]. The BMAA/HCO_3_^−^ interplay is likely to form a molecule structurally related to glu, acting as an agonist of its receptors (Figure 2), particularly ionotropic NMDA receptors (18). Other neurotoxicity experiments on cortical neurons have shown that BMAA binds to NMDA and non-NMDA glu receptors [19].

Pharmacological data have outlined this dependence to bicarbonate: the binding of glu to synaptic rat brain junctions is inhibited by BMAA (IC_50_ 1 mM) but only in the presence of HCO_3_^−^ (20 mM) [20]. This discovery made it possible to break a mechanistic lock and understand negative results generated on freshly isolated tissues bathed in a bicarbonate-free medium. The presence of HCO_3_^-^ ions at physiological pH thus allows the formation of carbamate adducts. At 37 °C and pH 7.4, BMAA reacts with HCO_3_^−^ to produce a β-carbamate, whose neurotoxicity is due to its structural homology to glu [21]. In vitro specific binding studies show that the toxin acts at 1 mM and always in the presence of physiological concentrations of bicarbonate (25 mM), by targeting ionotropic (NMDA) and metabotropic glu receptors of hippocampal slices of rat brains [22]. The specificity of BMAA for a sub-type of glu receptors is, therefore, relative, and other studies have proposed that its agonist activity could be simultaneously exerted on different classes, after intracerebroventricular injections of BMAA (500 μg/day) for 60 days in rats [23]. Consequently, BMAA acts on intracellular Ca^2+^ homeostasis: in brain cells of newborn rats, the toxin (5 mM) induced a very small Ca^2+^_i_ increase, which became important when HCO_3_^−^ is added to the extracellular medium [24]. These data confirm the central role of HCO_3_^−^ in the mechanism of action of BMAA and its metabolism, as well as the destabilization of Ca^2+^ homeostasis, a prelude to pro-apoptotic phenomena. In fact, as neurodegenerative disorders imply neuron mortality, the toxicity of BMAA was tested on motoneurons in vivo: intracranial injections in the striatum of mice show that BMAA (10 μL at a concentration of 100 mM) induces hippocampal neuron death in situ. These results are confirmed in vitro because BMAA exerts a dose-dependent cytotoxic effect (50 μM to 1 mM) on a spinal cell line close to motoneurons [25]. More recently, prolonged intrathecal injections of BMAA (5 mM) resulted in motoneuron degeneration in wild-type rats and in a similar manner to superoxyde dismutase 1 mutant (SOD1) rats [26]. SOD1 rodents are experimental models of human ALS. In vitro, BMAA at relatively low concentrations (10 μM) potentiates neuronal death induced by other neurotoxic molecules (NMDA, kainate, amyloid-β, 1-methyl-4-phenylpyridinium ions). This observation suggests that BMAA is likely to act at low concentrations as a co-actor of a neurodegenerative phenomenon involving other molecules. Finally, at moderate to high concentrations (30 μM to 1 mM), BMAA induces motoneuron death in vitro and this can be inhibited by the addition of an AMPA/kainate receptor antagonist. The BMAA-induced Ca^2+^ rise goes with the generation of reactive oxygen species in motoneurons, all these effects being much lower in other spinal neurons [27]. This interaction of BMAA with glutamatergic transmission was also tested on invertebrate neurons (the leech *Haemopis sanguisuga*) where the toxin (0.1 mM to 1 mM) induces a dose-dependent membrane depolarization of nerve-cell ganglia. Consequently, an increase in intracellular Na^+^ concentration and a decrease in intracellular K^+^ concentration occur. These data suggest that BMAA could initiate excitotoxic mechanisms by activation of other non-NMDA ionotropic receptors [28]. All these data point out that the toxin acts in vitro on neurons by mechanisms involving overactivation of glu receptors (Figure 2). This agonist interaction with ionotropic glu receptors is observed with domoic acid, an excitotoxin implicated in acute “amnesic shellfish poisoning” in humans and wildlife, but the long-term effects of which are not known [29].

## 3. Intracellular Actors Involved in BMAA-Induced Neurodegeneration

Some neurodegenerative processes involve recurrent biological markers, including hyperphosphorylated Tau protein (Tubulin-associated unit) and TDP-43 (TAR DNA-binding protein 43). Both have been widely challenged in cell assay with BMAA. The neuronal Tau is associated with microtubules and has been identified as the major component of the pairs of helical filaments (PHF) that constitute tangle degeneration, presumed to cause AD. In fact, the Tau protein of tangle degeneration are aggregated and abnormally phosphorylated [30]. Various studies have shown that BMAA interferes with the level of phosphorylation of the Tau protein, either by inhibiting the activity of its main phosphatase PP2A, or by increasing the activity of GSK3β kinase [31,32,33]. As we have seen before, BMAA also acts as an agonist of metabotropic glu receptors such as mGluR5 [33,34]. This activation in primary cultures of cortical neurons leads to a sharp decrease in the activity of PP2A. Indeed, activation of mGluR5 by BMAA caused a dissociation between PP2A and mGluR5, followed by phosphorylation of PP2A on Tyr307 by a Src family kinase. The decrease in PP2A phosphatase activity resulted in a higher level of Tau hyperphosphorylation. In the brains of the Guam cohort with ALS/PD syndrome, the catalytic activity of PP2A has been significantly reduced [33]. Furthermore, BMAA drives an increase in GSK3β synthesis and kinase activity, one of the targets of which is the Tau protein [31,32]. The toxic effects of BMAA is impaired by the inhibition of GSK3 activity [35,36,37]. These results were reproduced with chemical inhibitors of GSK3 and natural molecules such as sphingosine-1 phosphate or isolecaronic acid (Figure 2). 

TDP-43 (TAR DNA-binding protein 43) is a protein encoded by the *TARDBP* gene, located in the cell nucleus of most tissues. It binds itself to DNA and participates in the regulation of the transcription. It can also bind to RNA to ensure its stability. By cutting and rearranging the mRNAs by alternative splicing, TDP-43 controls the production of different versions of certain proteins [38]. At least 60 mutations in the *TARDBP* gene have been identified in patients with ALS [39,40,41,42]. Most of these mutations affect the region of the protein involved in the maturation of mRNA, which disrupts the production of other proteins. TDP-43 misfolding leads to the formation of protein aggregates of motoneurons in some ALS patients. It is not clearly established whether these aggregates cause the death of neuronal cells leading to ALS, or if they are a by-product of a dying cell. Some patients with ALS caused by mutations in the *TARDBP* gene also develop frontal-temporal dementia (FTD), which is a progressive brain disorder affecting personality, behaviour and language [42]. Individuals who develop both conditions are diagnosed as having ALS-FTD. Several in vitro and in vivo studies suggest that BMAA results in the overexpression of TDP-43 and the formation of protein aggregates [26,31,32,43,44]. Human neuroblastoma cultured with L-BMAA (10 mM during 24 h) accumulated truncated forms of TDP-43 (C-terminal fragments), phosphorylated and high molecular weight forms of this protein. These specific forms of TDP-43 are present in patients with ALS and FTD [31] and in the cerebellum and hippocampus of rats treated with intrathecal infusion of BMAA, as well as in the spinal cord where mild accumulation and aggregation of TDP-43 in the cytosol of some injured and degenerating motor neurons were observed [26].

## 4. Protein Incorporation of BMAA and Cellular Stress

Another hypothesis regarding the mechanism by which BMAA would exert its neurotoxic effects is its putative ability to get incorporated into proteins during protein synthesis [45,46]. l-BMAA can be transported into cells by the membrane X_c_^−^ system, an antiporter that mediates uptake of cystine into cells in exchange for exporting glu from the cell [47]. It incorporates protein and substitutes with ala and ser residues, but the toxin might also be associated to proteins through non-covalent bonds [45,48]. An in vivo study with s.c injection of ^14^C-labelled BMAA revealed the presence of the toxin in liver and CNS after 24 h [46]. The concentrations of BMAA found in tissues were comparable between the liver and the hypothalamus but significantly lower in the pituitary gland. A part of the toxin incorporates the hepatic protein fraction, but at much lower levels in the hypothalamus and pituitary gland (1–10% of hepatic rates as a function of injected doses of BMAA). On the other hand, after 28 weeks, no trace of BMAA could be detected in the adult rat liver and various CNS structures tested (hypothalamus, hippocampus, pituitary, putamen), suggesting a toxin clearance from these structures during this period. The protein incorporation of BMAA could result in protein-refolding defects, and a subsequent accumulation of misfolded proteins in lysosomes [48]. This protein-synthesis anomaly associated with a massive Ca^2+^ entry is also supposed to lead to cell stress at the endoplasmic reticulum (ER), deregulation of the reduction/oxidation systems (“redox”), and an activation of some pro-apoptotic caspases like caspase-12 and, thus, cell death [17,25,31,49,50,51,52,53,54,55]. A recent study has shown that the SOD1 protein (whose mutations are responsible for family forms of ALS), even lacking its dismutase activity (ApoSOD1), prevents the neurotoxic effects of BMAA on cultured NSC-34 motoneurons by activating a Ca^2+^/Akt/Erk kinase-dependent pathway [56]. These results are of interest as they also showed that SODG93A, a mutation associated with familial ALS, failed to protect motoneuron apoptosis induced by BMAA exposition. Genetic factors may also be involved in the neuropathogenesis induced by exposure to BMAA (Figure 2). 

Finally, a recent proteomic study has shown that the exposure of zebrafish embryos to BMAA induces an increase in the synthesis of proteins associated with the signalling of glutamate receptors as well as in protein homeostasis, oxidative metabolism and neuronal death [57]. These results were confirmed by another proteomic study in NSC-34 cells exposed for 72 h to BMAA (500 µM). Indeed, exposure to BMAA resulted in modification of several molecular pathways known to be implicated in ALS pathogenesis, such as ER stress, eIF2 signalling, protein ubiquitination and misfolded protein response pathways [58]. Interestingly, the expression of various mitochondrial proteins involved in oxidative phosphorylation, TCA cycle and oxidative stress response was found to be perturbed: BMAA favours activation of transcription factors known to regulate oxidative stress and cellular senescence [58]. Whereas these authors aimed to investigate the hypothesis that BMAA could be misincorporated into cellular protein in place of l-Serine, no evidence of BMAA misincorporation in intracellular or secreted proteins was found. Together, these studies demonstrated that BMAA, even at low concentrations, induces a dysregulation of the cellular protein homeostasis. However, there is no consensus on the hypothesis of protein incorporation of the BMAA, and further studies will have to be conducted to confirm or disprove it. 

## 5. Interaction of BMAA with Neuromelanin

The specificity of the Guam Island syndrome implies that BMAA or one of its metabolites (i) crosses the BBB and (ii) targets the structures involved in the control of voluntary movements, particularly the substantia nigra (SN), a cerebral area named for its dark pigment termed neuromelanin [59]. The bioavailability of BMAA in the CNS is still controversial, due to the technical complexity of efficiently measuring its amount [60,61] and BMAA analysis in human brains is contradictory [15,62,63]. Melanin and neuromelanin are concentrated in a few structures of the human CNS, including locus coeruleus, SN and retinal pigment epithelium (RPE). In the SN, it is a convenient biomarker to monitor, in medical imaging, the evolution of dopaminergic neuron death associated to PD [64], and neuromelanin defect is one of the current hypotheses for the development of PD [65]. For instance, it has been shown that some toxic compounds known to induce a parkinsonian syndrome such as 1-methyl-4-phenyl-1,2,9,6,6-tetrahydropyridine (MPTP) bind to neuromelanin and form a neurotoxic complex [66]. In addition, the antimalarial chloroquine competes with MPTP for binding to neuromelanin and protects intoxicated monkeys from the onset of parkinsonian disorders [67]. As MPTP, BMAA would bind melanin in various tissues including the CNS [68]. Twelve days after radiolabelled BMAA i.v injection in mice (0.91 µg/kg), a significant amount is detectable in the RPE indicating retinal blood-barrier crossing and CNS contamination. This BMAA accumulation in the pigmented tissues is not observed in albino mice, reflecting a possible interaction of BMAA with melanin present in the RPE cells of pigmented animals. BMAA has been shown to bind to melanin in a biphasic mode, suggesting two binding sites with different affinities. Since the mouse is devoid of neuromelanin in the SN, the authors compared it with an amphibian model. Radio-labelled BMAA was monitored on euthanized frogs (7.3 μg/kg body weight, s.c) and mice (7.3 μg/kg body weight, i.v) over 12 days. A toxin accumulation is correlated with the presence of neoformed neuromelanin in the tissues studied, suggesting a high storage of BMAA in pigment-rich cells. This capacity would correlate to the various clinical conditions observed in Guam patients including PD and pigmentary retinopathy [68]. 

**Figure 3:**
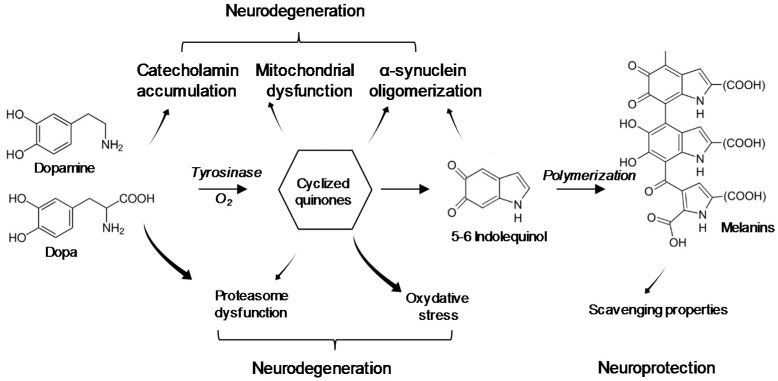
Origin and functions of melanins in the central nervous system. Melanin accumulates in catecholaminergic neurons and retinal pigmented epithelial cells from catecholamines (dopamine in substantia nigra and noradrenaline in locus ceruleus) and dopa derivates respectively. Intraneuronal neuromelanin could play a protective role during its synthesis by preventing the toxic accumulation of cytosolic catecholamines and derivatives. Those can trigger proteosomal (dopamine and *o*-quinones) and mitochondrial (*o*-quinones) dysfunctions, oxidative stress reactions (*o*-quinones) and α-synuclein oligomerization (*o*-quinones and 5–6 indolquinol). All those events can ultimately lead to the neuronal death and development of a neurodegenerative process. Melanin polymers limit the accumulation of catecholamines and derivates, therefore fighting neuronal death. In addition, by its ability to scavenge reactive metals, pesticides and other toxins to form stable adducts, this pigment also limits the extent of neurotoxic insults and provide neuroprotection to CNS areas expressing it.

Mechanistically, the binding of BMAA to neosynthesized melanin could lead to an alteration of the melanin polymer, making it less effective for trapping heavy metals or more sensitive to MPTP degradation [65,69,70]. In the brain, contrary to the skin and RPE, melanin synthesis does not involve any tyrosinase [71]. The auto-oxidation process of catecholamines is supposed to be a major source of neuromelanin [72]. The substrate involved in this oxidative polymerization is either norepinephrine in the locus coeruleus or dopamine in the SN and most other brain areas [73,74], this latter being an essential neurotransmitter for the striatal control [75]. The synthesis of neuromelanin could then prevent the accumulation of dopamine-oxidation products such as cyclized quinones (DA *o*-quinone, aminochrome and 5,6-indolequinone). Melanogenesis is considered a neuroprotective mechanism to fight accumulation of these highly toxic endogenous compounds (Figure 3). BMAA might disturb this pathway, thus leading to an excess of metabolites from the catabolism of catecholamines. These metabolites are involved in oxidation processes inducing mitochondrial dysfunction, free-radical accumulation, lipid peroxidation, protein degradation alteration and α-synuclein aggregation to neurotoxic oligomers, leading to an early neuronal aging common to several neurodegenerative pathologies [76,77,78,79,80]. Through the interaction with this pigment, and even without any incorporation into proteins, BMAA could be stored for a long period of time and be released throughout the life-time, which can lead to damage to the brain, notably a chronic inflammation and a subsequent gliosis contributing to the development of neurodegenerative disorders. In this slow kinetic model, neuromelanin would allow the storage of BMAA in precise brain structures where its rising concentrations could become neurotoxic to neuromelanin-rich neurons and neighbouring tissues upon BMAA release (Figure 3). This hypothesis explains: (i) the non-correlation between plasma and brain BMAA levels, as many studies have been carried out on neuromelanin-free brain areas; (ii) the original neurotoxicity of BMAA—as described in the Guam syndrome—including PD, ALS, AD and rare pigmentary retinopathy; and (iii) the very long-term impact of BMAA on exposed populations.

**Figure 4:**
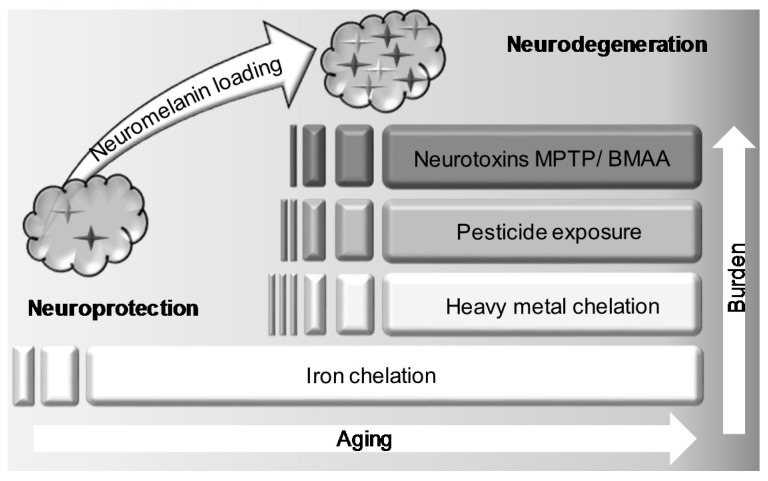
The Janus face of neuromelanin. Metal chelation by neuromelanin occurs throughout life in the CNS. It plays a protective role against iron cation accumulation and noxious consequences of heavy-metal exposure (Cd, Ch, Cu, …). Some pesticides components are known to interact with neuromelanin and finally neurotoxins MPTP and BMAA can bind to this neuronal pigment. Neurodegeneration may occur once burden cannot be fully handled by neuromelanin, letting free highly reactive compounds to accumulate in neuronal cytoplasm, causing neuronal death. Heavily charged melanosomes liberated from dead neurons trigger further inflammatory signals and may also contribute to a neurotoxic environment leading to the dreadful evolution towards neuropathology. There might be a pivotal point beyond which the situation cannot be restored to normal and neuronal survival is jeopardized. This point could be linked to individual neuromelanin stock, type of toxin accumulation, and aging status.

The hypothesis of BMAA sequestration is also in accordance with the dual role of neuromelanin in the CNS. This pigment interacts with many neurotoxic compounds, sequestering iron, toxic metals, agrochemicals [81,82], drugs [83] and neurotoxins [66], protecting the neurons from subsequent damage (Figure 4). However, the saturation of neuromelanin due to an overload of toxins may trigger a noxious cascade of events in which BMAA may be involved. When neuromelanin becomes saturated, free neuronal iron that cannot be handled any more by the pigment increases to the point where it starts to catalyze the production of free radicals [84,85]. In addition, hydrogen peroxide can degrade neuromelanin [86]. Pigmented neurons lose their protective agent and release iron, cytotoxic metals and other neurotoxic compounds accumulated over years that would accelerate neuronal death. Released pigment from dying cells in case of PD or neurotoxin intoxication [87] would be phagocytosed by glial cells and may trigger astrocytic and microglial activation via chemotactic effects and activation of pro-inflammatory factors. This could initiate a chronic inflammatory process in the affected areas [88]. Specific neurotoxin expositions, together with aging and individual stock of neuromelanin, will be negatively combined to reach a pivotal point beyond which the noxious sequence of events will ultimately lead to nigrostriatal pathologies: neurotoxic factors release, neuronal death, local neuro-inflammation, and massive neurodegeneration in SN. Sequestration of BMAA by melanin pigment could slowly and silently impair neuromelanin properties. Depending on the amount of exposure and the “burden status” of melanins, symptoms of BMAA intoxication could appear rapidly and last for years after exposure, as seen for MPTP intoxication [87,89]. 

## 6. Conclusions and Future Directions

BMAA induces neurotoxic effects, with multiple mechanisms. It certainly crosses the BBB to reach the CNS. At low concentrations, BMAA acts as an agonist of both iGluR and mGluR in vitro, resulting in an increased stimulation of motoneurons, leading progressively to Ca^2+^-dependent mechanisms of cellular apoptosis. The excitotoxic hypothesis, widely claimed in vitro, may find its limitation in the high concentrations necessary to reproduce effects in vivo [90] and in the difficulties to assess chronic excitotoxicity in vitro [91]. One of the future challenges will be to intoxicate neuronal cell models over long periods of time with low concentrations of BMAA, for parallel slow kinetics of degenerative phenomena.

It is also suggested that BMAA incorporates neuronal proteins, and substitutes for ala or ser residues, potentially inducing protein misfolding, aggregation mechanisms and cellular apoptosis [45]. But this hypothesis is controversial and recent in vitro data propose that BMAA does not misincorporate into proteins, adding to the complexity of its precise mode of action [92]. However, if confirmed, the incorporation of the toxin in proteins might explain its toxic bioaccumulation in cells coupled with a progressive release of BMAA in the CNS over the years, as a function of the protein turnover. Indeed, it is well established that incorporation of canonical amino acids at as low as 1/10,000 can lead to experimental neurodegeneration in vivo [93]. This BMAA exhibits proteic incorporation in various human cell lines, including fibroblasts, neuroblastoma and endothelial cells [48]. But this hypothesis is controversial in vivo, as chronic experiments have shown over several weeks the low and early incorporation of the toxin in liver and brain proteins and its long-term absence in such tissues [46]. BMAA also appears to induce an increase of several neurodegenerative-disease biomarkers, such as the hyperphosphorylated Tau protein and the presence of neurofibrillary plaques (Parkinson syndrome) or the aggregated form of TDP-43 (ALS). The toxin induces an increase in the hyperphosphorylated Tau protein, via the decrease in phosphatase 2, following the activation of mGluR5. It would be of interest to assess and follow the protein misincorporation of the toxin in intoxicated animals, and characterize—using proteomic approaches—the identity of BMAA-highjacked proteins. This point is an important challenge to better understand the mechanism by which BMAA promotes neuronal death. The issues will answer if BMAA misincorporation in proteins such as TDP-43 leads to their aggregation, and if BMAA misincorporation in the place of ser mimics protein-constitutive phosphorylation and hyperactivates signalling pathways.

Eventually, BMAA interacts with neuromelanin, a pigment present in dopaminergic networks of the brain and in the retina, leading to a long-lasting neurotoxic activity, compatible with the development of a neurodegenerative disease. In this hypothesis, it may be of interest to: (i) focus on melanin- and neuromelanin-rich structures, including locus coeruleus, SN and retinal tissue, for BMAA quantification in the CNS of ALS-dead patients; (ii) to use animal models with neuromelanin-containing tissues [94] and to avoid the murine model, which is almost devoid of cerebral neuromelanin [95], for toxicological assessments. These inter-species differences in the local expression of neuromelanin could, thus, provide an early explanation for contradictory studies on the effect of BMAA in the brain when comparing mouse (low neuromelanin content) and simian model (high neuromelanin content); (iii) to monitor the retinal function of persons or animals exposed to BMAA. Indeed, work on various animal models suggests that the retina appears to be a target of BMAA [51,96,97]. In humans, pigment retinopathy was identified in more than 50% of patients of Guam, who also had PD/AD [98,99,100,101]. This rare disease has also similarities with the syndrome induced by chloroquine overdose [102]. The classical tests for the functional exploration of vision (electroretinogram, optical-coherence tomography, fundus of vision, angiography, etc.) make it possible to detect precisely physiological damage, which could constitute rapid and non-invasive means of testing populations exposed to BMAA [103]. The co-occurrence of two rare conditions in the same patient (ALS and retinopathy) could thus be characteristic of exposure to BMAA and allow the discrimination of these cases from the other sporadic ALS observed in the global population. 

In conclusion, if BMAA cannot be considered as the cause of ALS or related neurological conditions; the hypothesis that it is a factor favouring neurotoxic phenomena is compelling, mainly because of its versatility in activating mechanisms at work in several neurodegenerative pathologies.
